# Aerobic Exercise Training Protects Against Insulin Resistance, Despite Low-Sodium Diet-Induced Increased Inflammation and Visceral Adiposity

**DOI:** 10.3390/ijms251810179

**Published:** 2024-09-22

**Authors:** Vanessa Del Bianco, Guilherme da Silva Ferreira, Ana Paula Garcia Bochi, Paula Ramos Pinto, Letícia Gomes Rodrigues, Luzia Naoko Shinohara Furukawa, Maristela Mitiko Okamoto, Jaíne Alves Almeida, Lizandre Keren Ramos da Silveira, Aritania Sousa Santos, Kely Cristina Soares Bispo, Vera Luiza Capelozzi, Maria Lucia Correa-Giannella, Alexandre Alves da Silva, Ana Paula Pereira Velosa, Edna Regina Nakandakare, Ubiratan Fabres Machado, Walcy Paganelli Rosolia Teodoro, Marisa Passarelli, Sergio Catanozi

**Affiliations:** 1 Laboratorio de Lipides (LIM-10), Hospital das Clinicas (HCFMUSP) da Faculdade de Medicina da Universidade de Sao Paulo, Sao Paulo 01246 000, Brazil; vdelbianco84@gmail.com (V.D.B.); ferreira.gui@hotmail.com (G.d.S.F.); bochianapaula@gmail.com (A.P.G.B.); paularamos83@gmail.com (P.R.P.); letigrodrigues1@gmail.com (L.G.R.); enakonda@usp.br (E.R.N.); m.passarelli@fm.usp.br (M.P.); 2 Laboratory of Renal Pathophysiology, Department of Internal Medicine, School of Medicine, University of São Paulo, São Paulo 01246 000, Brazil; luzia@usp.br; 3 Department of Physiology and Biophysics, Institute of Biomedical Sciences, University of São Paulo, São Paulo 05508 000, Brazil; mokamoto@icb.usp.br (M.M.O.); ubiratan@icb.usp.br (U.F.M.); 4 Rheumatology Division of the Hospital das Clinicas, University of São Paulo Medical School, São Paulo 01246 000, Brazil; jaine_alves_almeida@outlook.com (J.A.A.); lizandre_keren@usp.br (L.K.R.d.S.); apvelosa@gmail.com (A.P.P.V.); walcy.teodoro@fm.usp.br (W.P.R.T.); 5 Laboratorio de Carboidratos e Radioimunoensaios (Laboratorio de Investigações Médicas, LIM-18), Faculdade de Medicina, Universidade de Sao Paulo (FMUSP), Sao Paulo 01246 000, Brazil; aritania@alumni.usp.br (A.S.S.); maria.giannella@fm.usp.br (M.L.C.-G.); 6 Department of Pathology of the Hospital das Clinicas da Faculdade de Medicina da Universidade de Sao Paulo, FMUSP, Sao Paulo 01246 000, Brazil; kelybispo@hotmail.com (K.C.S.B.); vera.capelozzi@fm.usp.br (V.L.C.); 7 Department of Physiology and Biophysics, Mississippi Center for Obesity Research, Cardiorenal and Metabolic Diseases Research Center, University of Mississippi Medical Center, Jackson, MS 39216, USA; asilva@umc.edu; 8 Programa de Pós Graduação em Medicina, Universidade Nove de Julho, Sao Paulo 01525 000, Brazil

**Keywords:** dietary sodium restriction, insulin resistance, dyslipidemia, aerobic exercise training, adipose tissue

## Abstract

Dietary sodium restriction increases plasma triglycerides (TG) and total cholesterol (TC) concentrations as well as causing insulin resistance and stimulation of the renin-angiotensin-aldosterone system (RAAS) and the sympathetic nervous system. Stimulation of the angiotensin II type-1 receptor (AT1) is associated with insulin resistance, inflammation, and the inhibition of adipogenesis. The current study investigated whether aerobic exercise training (AET) mitigates or inhibits the adverse effects of dietary sodium restriction on adiposity, inflammation, and insulin sensitivity in periepididymal adipose tissue. LDL receptor knockout mice were fed either a normal-sodium (NS; 1.27% NaCl) or a low-sodium (LS; 0.15% NaCl) diet and were either subjected to AET for 90 days or kept sedentary. Body mass, blood pressure (BP), hematocrit, plasma TC, TG, glucose and 24-hour urinary sodium (U_Na_) concentrations, insulin sensitivity, lipoprotein profile, histopathological analyses, and gene and protein expression were determined. The results were evaluated using two-way ANOVA. Differences were not observed in BP, hematocrit, diet consumption, and TC. The LS diet was found to enhance body mass, insulin resistance, plasma glucose, TG, LDL-C, and VLDL-TG and reduce U_Na_, HDL-C, and HDL-TG, showing a pro-atherogenic lipid profile. In periepididymal adipose tissue, the LS diet increased tissue mass, TG, TC, AT1 receptor, pro-inflammatory macro-phages contents, and the area of adipocytes; contrarily, the LS diet decreased anti-inflammatory macrophages, protein contents and the transcription of genes related to insulin sensitivity. The AET prevented insulin resistance, but did not protect against dyslipidemia, adipose tissue pro-inflammatory profile, increased tissue mass, AT1 receptor expression, TG, and TC induced by the LS diet.

## 1. Introduction

Several studies have shown a direct association between sodium intake and cardiovascular disease (CVD) mortality [1,2]. Conversely, low-sodium (LS) intake reduces blood pressure (BP) [3] and CVD mortality in normotensive and hypertensive individuals [4].

Although dietary sodium restriction reduces BP and prevents hypertension, previous studies show that a LS diet increases CVD risk factors including sympathetic activation and stimulation of the renin-angiotensin-aldosterone system (RAAS), peripheral insulin resistance [3,5], and enhanced plasma lipid concentrations [6,7]. Therefore, a J- or U-shaped association between sodium intake and cardiovascular mortality has been suggested [8,9]. Clinical and preclinical studies also report that dietary sodium chloride restriction elevates plasma lipids and induces arterial-wall lipid infiltration [3,6,7,10,11,12,13,14]. In dyslipidemic low-density lipoprotein receptor knockout (LDLR KO) mice, dietary-sodium restriction adversely changed lipid profile related to mitochondrial function in the gastrocnemius muscle, including increased glycerophospholipids and reduced cardiolipins, which are associated with development of insulin resistance [15]. LS intake also impairs liver insulin sensitivity, which is associated with increased expression of genes that modulate gluconeogenesis and lipogenesis and that inhibit lipoprotein lipase activity, such as apolipoprotein CIII [16].

In addition to the observations described above, LS intake is also associated with increased atherogenic potential as evidenced by enhanced lipid infiltration in the aortic arch and brachiocephalic trunk of mice fed a LS diet [14,17]. Compared to the wild-type, LDLR KO mice fed a conventional low-fat diet are moderately hypercholesterolemic and hypertriglyceridemic with a chronic low-intensity inflammatory state induced by the hyperlipidemia, which favors atherogenesis [14,17]. This mouse model of dyslipidemia facilitates the investigation of whether intense and chronic dietary sodium restriction adversely influences lipid and inflammatory profiles, insulin resistance, and atherogenesis [14,15,16,17]. Interestingly, the LS diet induced-atherogenesis was prevented by pharmacological antagonism of the RAAS, which reduced arterial wall glycoxidative stress independent of reductions in BP in hypertensive hyperlipidemic mice [14]. Wistar rats fed a LS diet (0.06% sodium) exhibit insulin resistance in liver, skeletal muscle, and adipose tissues, favoring increased adiposity [18].

Recently, it was shown that aerobic exercise training (AET) reduces lipid infiltration and markers of lipid peroxidation and glycoxidation in the brachiocephalic trunk of dyslipidemic mice fed a sodium restricted diet [17]. AET reduces cardiovascular mortality by modulating cardiometabolic risk factors, such as reducing BP, small dense low-density lipoprotein (LDL), and triglyceride (TG) levels, while increasing high-density lipoprotein cholesterol (HDL-C) and insulin sensitivity [19,20].

The current study investigated the impact of LS intake on visceral adipose tissue inflammation, plasma lipid profile, the content of proteins that mediate insulin signaling pathway, the adipocyte area as well as the modulation of these parameters by AET. Our findings show that chronic LS intake increased plasma TG levels, promoted insulin resistance and a pro-atherogenic lipoprotein (LP) profile, and increased the adipocyte area and inflammation in periepididymal adipose tissue. We also observed that AET protected against the development of insulin resistance but did not prevent inflammation or the increase in visceral adiposity induced by dietary sodium restriction.

## 2. Results

### 2.1. Baseline Parameters

Before starting the experiments (LS or NS diet intake and AET), we examined basal plasma glucose, TC, TG, hematocrit, and SBP in all groups (see Table 1), as previously reported [15,16,17].

### 2.2. Effects of Dietary Sodium Restriction and Aerobic Exercise Training on Plasma Lipids, Hematocrit, and Body Mass of LDLR KO Mice

**Figure 1:**
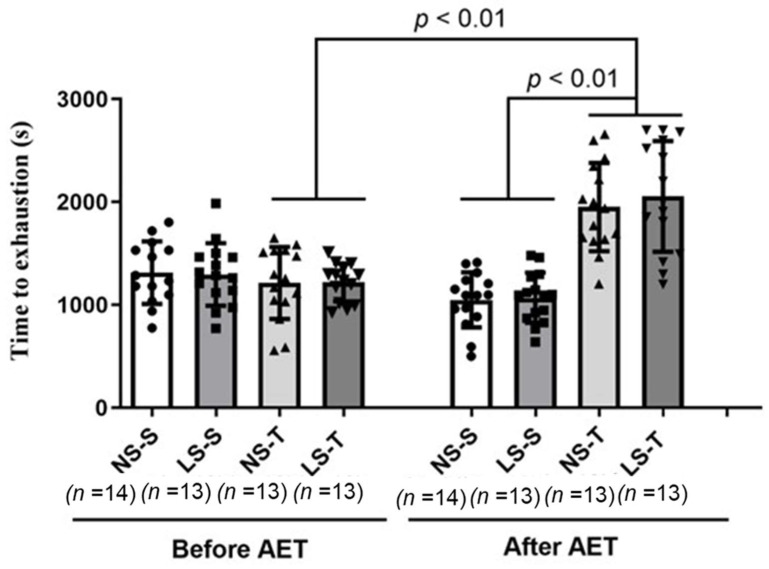
Maximal exercise capacity test in LDL receptor knockout mice chronically fed a low (LS) or normal sodium (NS) diet, trained (T) or sedentary (S). Results are expressed as mean ± standard deviation (SD) and compared by two-way ANOVA with Tukey’s post-test. *n* = number of mice. *p*-values represent the statistical differences between sedentary and trained groups over time (AET, aerobic exercise training × time).

After 90 days of the experimental intervention (administration of diets and AET), animals fed the LS diet showed an increase in BM, plasma glucose, and TG concentrations when compared to animals fed the NS diet. Plasma TC, hematocrit, and SBP were not different among the groups (see Table 2), as previously reported [15,16,17]. As expected, mice fed the LS diet and trained groups (NS-T and LS-T) exhibited lower 24-h U_Na_ (see Table 2) and higher maximum exercise capacity (see Figure 1), respectively, when compared to the NS diet and sedentary groups demonstrating the effectiveness of both interventions, as previously reported [16,17]. 

### 2.3. Normal and Low Sodium Diet Consumption 

**Figure 2:**
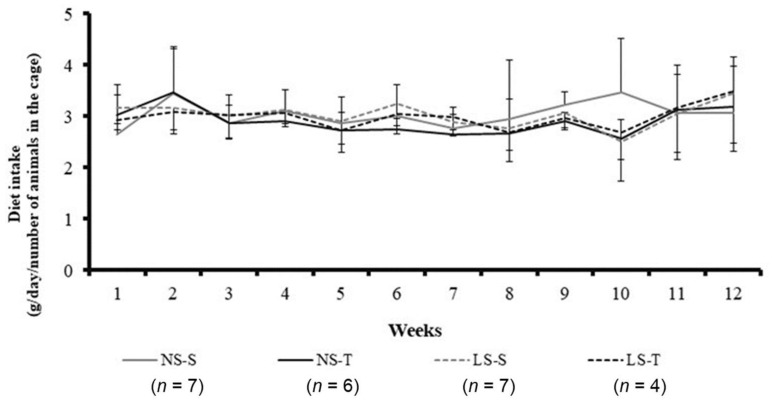
Normal (NS) and low-sodium (LS) diet intake in LDL receptor knockout mice during the 90-day experimental protocol (g/day/number of animals in the cage). Data represented as mean ± standard deviation (NS and LS groups with standard deviation upwards and downwards, respectively). Results compared by three-way ANOVA (random factor: animal; fixed factor: time, diet and exercise) with Tukey’s post hoc test. *p* exercise × time < 0.01; *p* exercise × diet × time = 0.32. S, sedentary; T, trained; *n* = number of mice.

Normal and low sodium diet consumption was similar among groups during the 90-day experimental protocol (see Figure 2), as previously reported [16,17].

### 2.4. Aerobic Exercise Training Protected against Peripheral Insulin Resistance Induced by Low-Sodium Diet 

**Figure 3:**
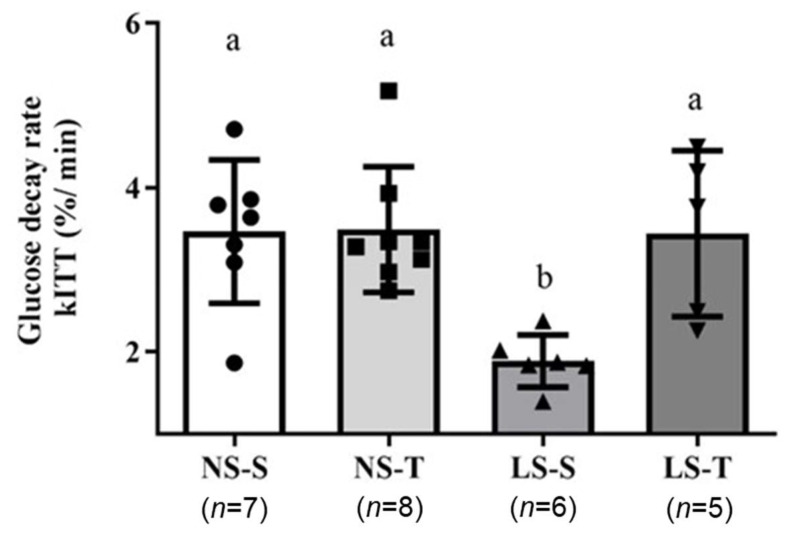
Insulin tolerance test in LDL receptor knockout mice chronically fed a normal (NS) or low-sodium (LS) diet, sedentary (S) or trained (T), after 90-day experimental protocol. The blood glucose decay rate (kITT) was calculated by linear regression between baseline and 30 min. Results were expressed as mean ± standard deviation (SD) and compared by two-way ANOVA (generalized linear model; fixed factors: diet and AET) with Tukey’s post-test. *n* = number of animals. Distinct letters represent statistical difference among groups (*p* < 0.05). AET, aerobic exercise training.

During the insulin tolerance test (ITT), the blood glucose decay rate was slower in LS–S mice when compared to the other groups, as previously reported [16,17], suggesting peripheral insulin resistance elicited by the dietary sodium restriction, which was prevented by the AET (LS–T group) (Figure 3).

### 2.5. Low-Sodium Diet Induced an Atherogenic Lipoprotein Profile 

**Figure 4:**
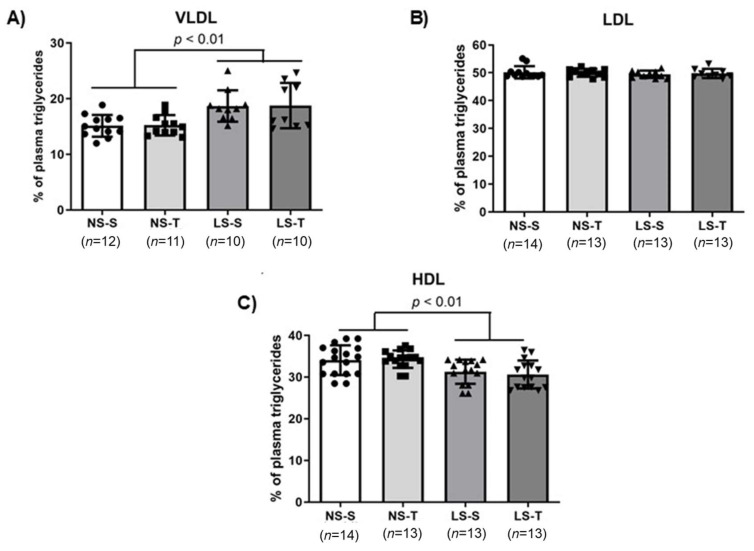
Triglycerides (TG) percentage in lipoprotein fractions ((**A**), VLDL; (**B**), LDL; (**C**), HDL) in LDL receptor knockout mice chronically fed the normal (NS) or low-sodium (LS) diet, sedentary (S) or trained (T), after 90-day experimental protocol. Results are expressed as mean ± standard deviation (SD) and compared by two-factor ANOVA (generalized linear model; fixed factors: diet and AET) with Tukey’s post-test. *n* = number of animals. AET, aerobic exercise training.

**Figure 5:**
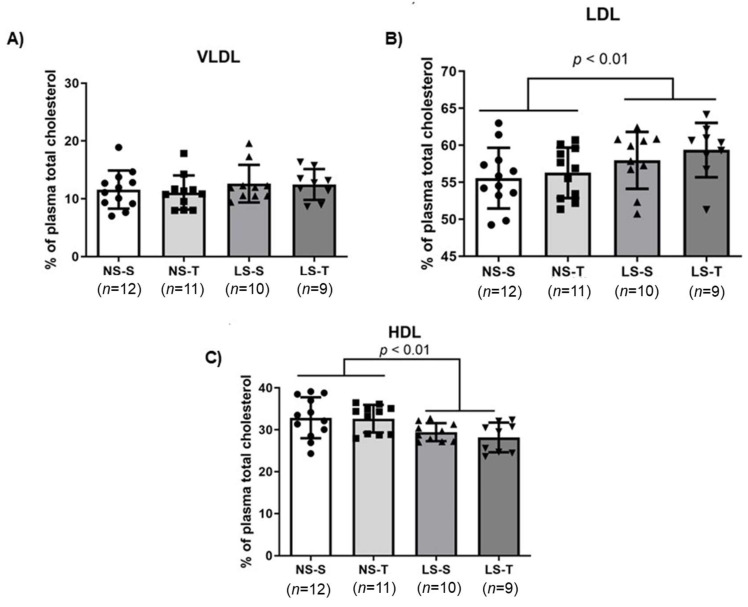
Total cholesterol (TC) percentage in lipoprotein fractions ((**A**), VLDL; (**B**), LDL; (**C**), HDL) in LDL receptor knockout mice chronically fed the normal (NS) or low-sodium (LS) diet, sedentary (S) or trained (T), after 90-day experimental protocol. Results are expressed as mean ± standard deviation (SD) and compared by two-factor ANOVA (generalized linear model; fixed factors: diet and AET) with Tukey’s post-test. *n* = number of animals. AET, aerobic exercise training.

Chromatographic analysis of plasma LP showed that animals fed the LS diet had a higher and lower TG percentage for VLDL and HDL (Figure 4A,C), respectively, compared to the NS diet groups. The TG percentage in the LDL was not different among animals (see Figure 4B), as previously reported [16,17]. The LS diet also increased and reduced cholesterol percentage in LDL and HDL (Figure 5B,C), respectively, compared to the NS diet groups. The cholesterol percentage in the VLDL was not different among animals (see Figure 5A), as previously reported [16,17]. 

### 2.6. Low-Sodium Diet Increased Periepididymal Adipose Tissue Mass

**Figure 6:**
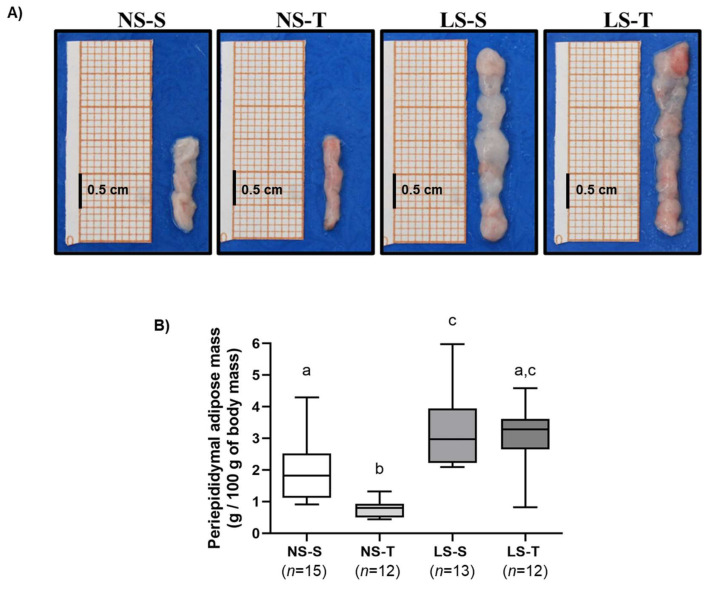
Periepididymal adipose tissue of LDL receptor knockout mice chronically fed a normal (NS) or low-sodium (LS) diet, sedentary (S) or trained (T), after 90-day experimental protocol. (**A**) representative images of periepididymal adipose tissue mass isolated from each experimental group (macroscopic view); (**B**) periepididymal adipose mass of the experimental groups. Results are expressed as median and range (maximum value–minimum value) and compared by the Kruskall–Wallis test with Dunn’s post-test. *n* = number of animals. Distinct letters represent statistical difference among groups (*p* < 0.05).

Periepididymal adipose tissue mass was assessed to examine the adipogenic effect of LS intake. Periepididymal adipose tissue mass was higher in the LS diet mice, when compared to the NS diet groups. AET reduced adiposity in the NS-T group, but did not prevent the increase in adipose mass induced by the LS diet (LS–S and LS–T groups) (see Figure 6A,B).

### 2.7. Low-Sodium Diet Increased TG and TC Content in Periepididymal Adipose Tissue

**Figure 7:**
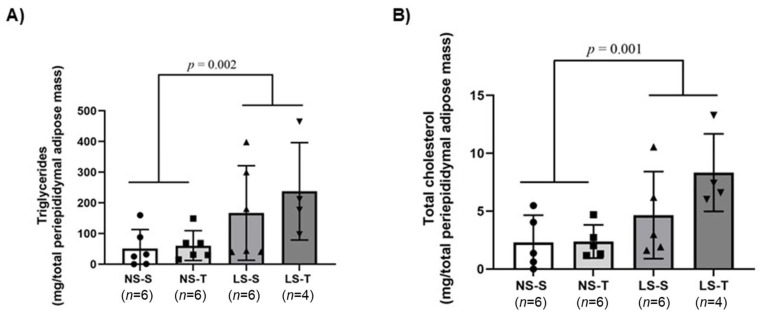
Periepididymal adipose tissue lipid concentration in LDL receptor knockout mice chronically fed a normal (NS) or low-sodium (LS) diet, sedentary (S) or trained (T), after 90-day experimental protocol. (**A**) triglycerides (TG) (mg/total periepididymal adipose mass); (**B**) total cholesterol (TC) (mg/total periepididymal adipose mass). Lipids were extracted from ~100 mg of periepididymal adipose tissue by the method of Folch [21] and lipid concentrations were determined by colorimetric enzymatic kits. Results are expressed as mean ± standard deviation (SD) and compared by two-way ANOVA (generalized linear model; fixed factors: diet and AET) with Tukey’s post-test. *n* = number of animals. AET, aerobic exercise training.

The increased visceral fat mass in the LS diet groups prompted us to test whether LS intake increases periepididymal adipose tissue lipid content. TG and TC concentrations were higher in periepididymal adipose tissue excised from mice fed the LS diet when compared to the NS diet mice (Figure 7A,B). AET did not prevent the increase in adipose tissue lipid concentration induced by the LS diet.

### 2.8. Low-Sodium Diet Increases Individual Adipocyte Area in Periepididymal Adipose Tissue

**Figure 8:**
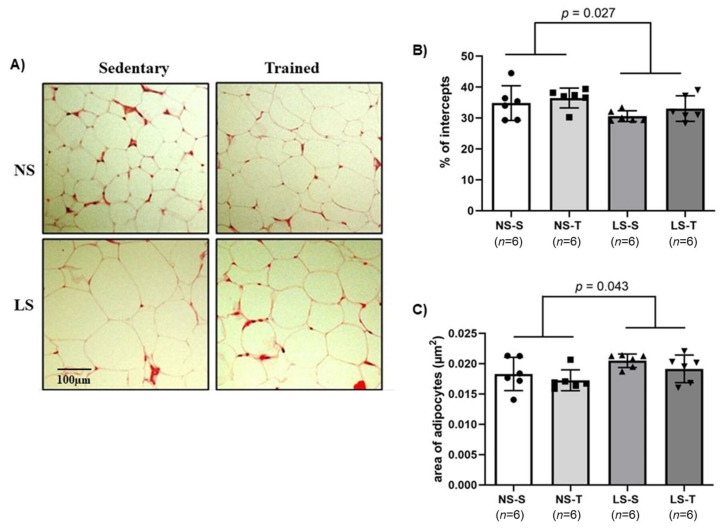
Periepididymal adipose tissue histomorphometry in LDL receptor knockout mice chronically fed a normal (NS) or low-sodium (LS) diet, sedentary (S) or trained (T), after 90-day experimental protocol. (**A**) representative micrographs of transverse histological sections (4 µm thick) of periepididymal adipose tissue, stained with hematoxylin and eosin. The images were digitized using 200× magnification; (**B**) histomorphometric analysis of the percentage of intercepts in periepididymal adipose tissue (see methods for details); (**C**) graph of estimated area of adipocytes in the periepididymal adipose tissue (see methods for details). Results are expressed as mean ± standard deviation (SD) and compared by two-factor ANOVA (generalized linear model; fixed factors: diet and AET) with Tukey’s post-test. *n* = number of animals. AET, aerobic exercise training.

As the lipid content in periepididymal adipose tissue was higher in the LS mice, we investigated if individual adipocyte area was also increased in mice fed a LS diet. We observed that mice fed the LS diet had a lower percentage of intercepts in the periepididymal adipose tissue (see Figure 8B), indicating a larger area of individual adipocytes when compared the NS diet mice (see Figure 8C). Furthermore, we found that AET did not prevent a reduction in the percentage of intercepts (e.g., increase in area of individual adipocytes) induced by the LS diet (see Figure 8B). These changes in the adipose tissue may reflect a larger size, a smaller number (see Figure 8A), and an unfavorable metabolic status of adipocytes in the LS mice.

### 2.9. Low-Sodium Diet Decreased the Expression of Genes Related to Insulin Sensitivity in Periepididymal Adipose Tissue

**Figure 9:**
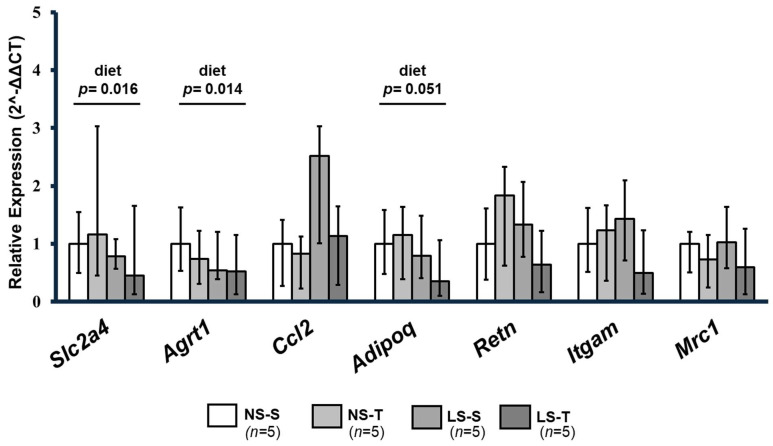
Relative gene expression (2^−ΔΔCT^) of genes involved in insulin signaling and sensitivity—GLUT4 (*Slc2a4*), adiponectin (*Adipoq*)—and in inflammatory mechanisms—angiotensin II receptor type-1 (*Agrt1*), integrin alpha M (*Itgam*), mannose C receptor type-1 (*Mrc1*), resistin (*Retn*), and chemokine (*C-C motif*) ligand 2 (*Ccl2*) in the periepididymal adipose tissue of LDL receptor knockout mice chronically fed a normal (NS) or low-sodium (LS) diet, sedentary (S) or trained (T), after 90-day experimental protocol. *Ppib* (*Peptidyl-prolyl cis-trans isomerase B*) was used as a reaction-normalizing gene. Gene expression was calculated by the formula: 2-((CT of the target gene—mean endogenous control CT)—calibrator), and the mean CT of the NS–S group as calibrator (2^−ΔΔCT^). Results presented as amplitude of expression and standard deviation were compared by two-factor ANOVA test with two fixed factors (diet and exercise) with Tukey’s post-test. *n* = number of mice. Data are expressed as mean ± range. Results were compared by 2-way ANOVA (GLM; fixed factor: diet and exercise) with Tukey’s post hoc test. “Diet” = *p* < 0.05 NS vs. LS. *n* = number of animals.

Consumption of a LS diet was associated with reduced gene expression of *Slc2a4* (encodes information for production of GLUT4), *Agrt1* (encodes information for production of AT1 receptor), and *Adipoq* (encodes information for production of adiponectin, which promotes insulin sensitivity and is anti-inflammatory). Other genes involved in inflammation (*Il10*, *Tnf*, *Itgam*, *Mrc1*, *Retn,* and *Ccl2*) were not changed by the diet or AET (see Figure 9).

### 2.10. Aerobic Exercise Training Protected against Reduction of GLUT4 Content in Periepididymal Adipose Tissue Induced by the Low-Sodium Diet 

**Figure 10:**
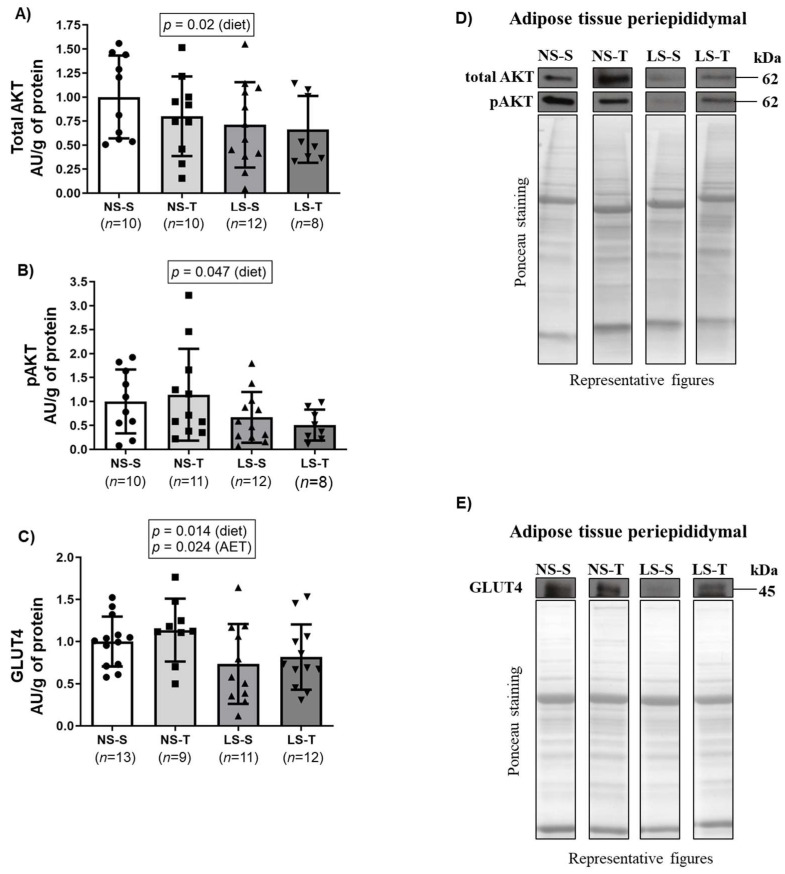
Protein quantification and representative Western Blotting images of the expression of total AKT (**A**,**D**), phosphorylated AKT (pAKT) (**B**,**D**), and GLUT4 (**C**,**E**) in the periepididymal adipose tissue of LDL receptor knockout mice chronically fed a normal (NS) or low-sodium (LS) diet, sedentary (S) or trained (T), after 90-day experimental protocol. Results are expressed as mean ± standard deviation (SD) and compared by two-way ANOVA (generalized linear model; fixed factors: diet and AET) with Tukey’s post-test. *n* = number of animals. AET, aerobic exercise training; AU, arbitrary units.

We examined the content of several insulin signaling proteins in periepididymal adipose tissue that could contribute to LS diet-induced peripheral insulin resistance, hyperlipidemia and the apparent unfavorable metabolic status of adipocytes in periepididymal adipose tissue (higher adipose tissue mass, lipid concentration, and individual adipocyte area). The LS mice exhibited lower content of total AKT, phosphorylated AKT, and GLUT4 (see Figure 10A–E) in adipocytes when compared to adipocytes from the NS diet mice. We also found that AET protected against the reduction of GLUT4 protein expression in periepididymal adipose tissue of mice fed the LS diet (see Figure 10C,E).

### 2.11. Low-Sodium Diet Promoted Inflammation in Periepididymal Adipose Tissue

**Figure 11:**
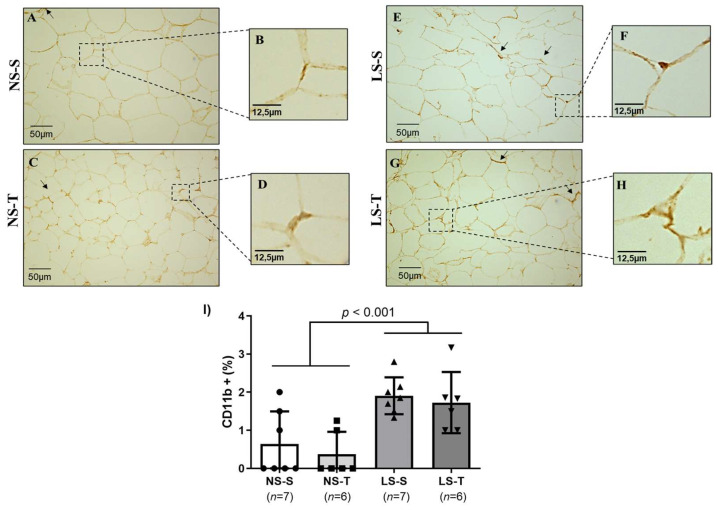
Immunostaining for CD11b+ macrophages (M1), pro-inflammatory subtype, in the periepididymal adipose tissue of LDL receptor knockout mice chronically fed a normal (NS) or low-sodium (LS) diet, sedentary (S) or trained (T), after 90-day experimental protocol. Representative micrographs (**A**,**C**,**E**,**G**) denote adipocytes with positive staining (brown staining) for subtype M1 macrophages. The periepididymal adipose tissue presents a homogeneous cytoplasmic pattern (400× magnification). The arrows in representative micrographs (**A**,**C**,**E**,**G**) show in detail (micrographs (**B**,**D**,**F**,**H**)) the positive cells of the respective groups: NS–S, NS–T, LS–S, and LS–T. (**I**) Graph representation of the percentage of CD11b+ cells in periepididymal adipose tissue. Results are expressed as mean ± standard deviation (SD) and compared by two-factor ANOVA (generalized linear model; fixed factors: diet and AET) with Tukey’s post-test. *n* = number of animals. AET, aerobic exercise training.

**Figure 12:**
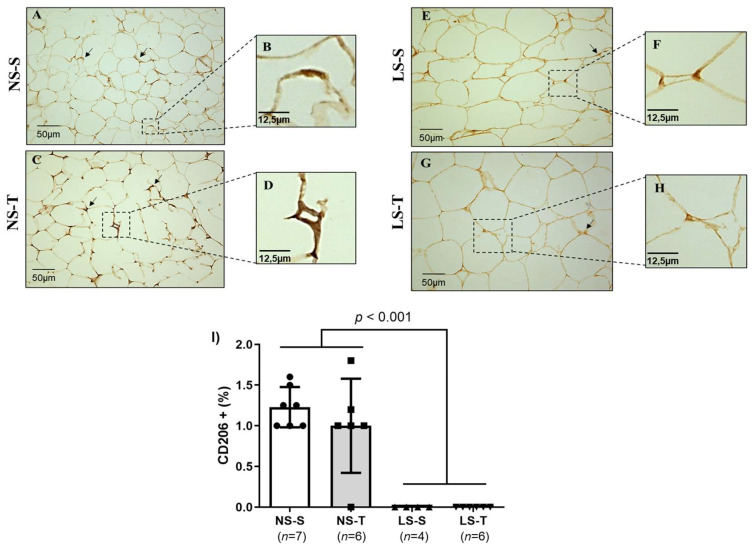
Immunostaining for CD206+ macrophages (M2), anti-inflammatory subtype, in the periepididymal adipose tissue of LDL receptor knockout mice chronically fed a normal (NS) or low-sodium (LS) diet, sedentary (S) or trained (T), after 90-day experimental protocol. Representative micrographs (**A**,**C**,**E**,**G**) denote adipocytes with positive staining (brown staining) for subtype M2 macrophages. The periepididymal adipose tissue presents a homogeneous cytoplasmic pattern (400× magnification). The arrows in the representative micrographs (**A**,**C**,**E**,**G**) show in detail (micrographs (**B**,**D**,**F**,**H**) the positive cells of the respective groups: NS–S, NS–T, LS–S and LS–T. (**I**) Graph representation of the percentage of CD206+ cells in periepididymal adipose tissue. Results are expressed as mean ± standard deviation (SD) and compared by two-factor ANOVA (generalized linear model; fixed factors: diet and AET) with Tukey’s post-test. *n* = number of animals. AET, aerobic exercise training.

We evaluated whether periepididymal adipose tissue inflammation was involved in the systemic and local metabolic disorders promoted by the LS diet. Compared to the NS diet groups (see Figure 11A–D and Figure 12A–D), the LS diet mice showed increased immunostaining for CD11b (marker of subtype M1 pro-inflammatory macrophage) (see Figure 11E–H) and reduced immunostaining for CD206 (marker of subtype M2 anti-inflammatory macrophage) (see Figure 12E–H), represented by a diffuse pattern and brown staining in the stromal area of the periepididymal adipose tissue. AET did not prevent the adverse macrophage profile in periepididymal adipose tissue of the LS diet mice (see Figure 11I and Figure 12I).

### 2.12. Low-Sodium Diet Increased AT1 Content in Periepididymal Adipose Tissue

**Figure 13:**
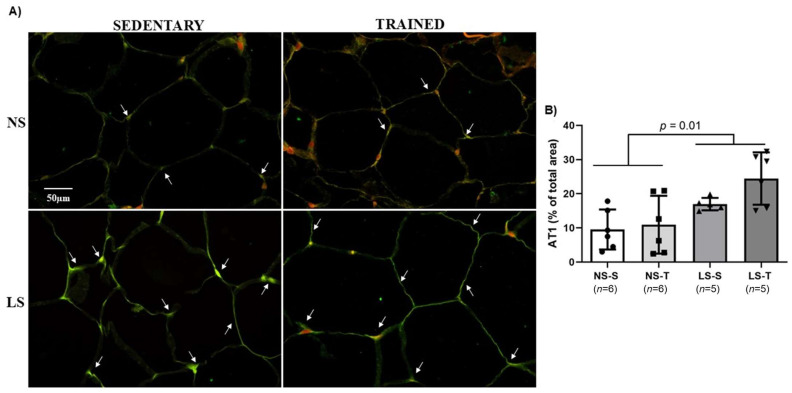
Angiotensin II type 1 receptor (AT1) in LDLR KO mice fed either a normal-sodium (NS) or a low-sodium (LS) diet, trained (T) or sedentary (S), after 90-day experimental protocol. (**A**) representative micrographs of immunofluorescence-stained AT1 in the periepididymal adipose tissue (400× magnification). The arrows in the representative micrographs indicate that the green immunostaining shows AT1 expression; (**B**) histomorphometric analysis of immunofluorescence-stained AT1 in periepididymal adipose tissue. Results are expressed as mean ± standard deviation (SD) and compared by two-factor ANOVA (generalized linear model; fixed factors: diet and AET) with Tukey’s post-test. *n* = number of animals. AET, aerobic exercise training.

Since activation of AT1 receptors favors adiposity, adipocyte hypertrophy, inflammation, and insulin resistance, we assessed the influence of the LS diet on AT1 receptor expression in periepididymal adipose tissue. The AT1 receptor content was higher in mice fed the LS diet compared to the NS diet mice (see Figure 13A). The AET did not protect against the increased AT1 receptor expression induced by the LS diet (see Figure 13B).

## 3. Discussion

It is challenging to select the most appropriate murine models to study the pathophysiology of dietary sodium restriction induced-dyslipidemia and insulin resistance. The advantages, disadvantages, and limitations of each model make it extremely difficult to accurately establish animal models for investigations of metabolic disorders reflecting insulin resistance, hyperlipidemia, and cardiovascular disease in humans [22]. Compared to the wild-type strain, LDLR KO mice fed a conventional diet (low in fat concentration) show moderate hypercholesterolemia (~6.5 mmol/L) that favors atherogenesis. The plasma LP profile reveals increased intermediate density lipoproteins (IDL) and LDL concentrations without changes in HDL content. Thus, unlike wild mice, which HDL is the main particle, in the LDLR KO mice the LDL is the major plasma lipoprotein, generating a lipid profile similar to humans. Therefore, the LDLR KO model shares features observed in human familial hypercholesterolemia [23,24,25].

In the present study, LDLR KO mice were chronically fed a NS or LS diet and subjected to AET or kept sedentary for 90 days. Peripheral insulin resistance, cholesterolemia, triglyceridemia, and LP profile were assessed. In the periepididymal adipose tissue, TG and TC concentration, the percentage of intercepts (an index of individual adipocyte area), tissue mass, macrophage polarization and the transcription of genes and expression of proteins related to insulin signaling were evaluated. We also examined the potential benefits of AET on glycolipid metabolism and in mitigating the adverse effects of LS diet on systemic lipid metabolism and insulin sensitivity as well as on periepididymal adipose tissue inflammation, adipocyte morphology, and gene and protein expression. Our findings reveal that the LS diet increased body mass, triglyceridemia, and blood glucose, impaired peripheral insulin sensitivity, and promoted a pro-atherogenic LP profile. SBP, hematocrit, and cholesterolemia were not different among experimental groups. In periepididymal adipose tissue, LS intake markedly increased tissue mass, pro-inflammatory macrophage (M1 subtype) infiltration, and TG and TC concentrations. Furthermore, we observed an enhanced percentage of intercepts (indicating increased individual adipocyte area) and a decreased content of anti-inflammatory macrophages (subtype M2) as well as proteins involved in insulin signaling and glucose uptake in periepididymal adipose tissue. Finally, we found that AET protected against the reduction in GLUT4 expression in periepididymal adipose tissue in mice induced by the LS diet.

The lower U_Na_ in mice fed the LS diet and the higher exercise capacity of the trained groups, when compared to the baseline values (a 60% increase) and to the sedentary groups at the end of the intervention (a 79% increase), show the effectiveness of both experimental interventions (the administration of diets and AET). The exercise intensity used in our study was not corrected during the 90-day experimental protocol. Running speed (15 m/min) corresponded to about 56% and 39% of the maximum speeds obtained in the maximum exercise capacity test, respectively, for the first week and at the end of the protocol. This indicates that we exposed the mice to a moderate-to-low intensity physical training.

Studies in humans and laboratory animals have shown that dietary sodium restriction unfavorably modulates lipid metabolism, evidenced by enhanced TC, TG, and non-esterified fatty acids (NEFA) plasma concentrations, which leads to a predisposition toward early arterial lipid infiltration [3,6,7,10,11,12,13,14,26]. Insulin resistance induced by dietary sodium restriction reduces lipoprotein lipase enzyme expression and activity, impairing TG-rich plasma LP (chylomicron and very low-density lipoprotein) metabolism and favoring hypertriglyceridemia [6]. Previously, we showed that increased phosphatidylcholines (68%), phosphatidylinositol (90%), and NEFA (59%) were associated with cardiolipins (41%) and acylcarnitines (9%) reduction in gastrocnemius muscle, which markedly favored peripheral insulin resistance induced by the LS diet in this experimental model [15].

In the present study, the LS diet reduced periepididymal adipose tissue protein content of total AKT, phosphorylated AKT, and GLUT4, contributing to peripheral insulin resistance, and this was prevented by AET. These results can be explained, at least in part, by the angiotensin II (ANG II) type 1 receptor (AT1) receptor stimulation. AT1 receptor activation has been shown to increase NADPH oxidase enzyme complex activity and reactive oxygen species generation [27]. The activation of redox-sensitive serine kinases, such as c-jun N-terminal kinase (JNK) and extracellular signal-regulated kinase (ERK)-1, leads to phosphorylation of serines in the insulin receptor substrate-1 (IRS-1) and decreases IRS-1 interaction with phosphatidylinositol 3-kinase (PI3K). Consequently, there is reduced AKT and protein kinase C (PKC) activation and impaired GLUT4 translocation to the cytoplasmic membrane favoring insulin resistance [28].

Although cholesterolemia was not different among groups, we showed that the LS diet increased LDL-C and reduced HDL-C, revealing a pro-atherogenic lipid profile and increased atherogenesis [17]. We observed that LS consumption increased both weight and periepididymal adipose tissue mass in the sedentary and trained groups. The increase in periepididymal adipose mass in the LS diet fed groups was also associated with a lower percentage of intercepts, indicating increased individual adipocyte area, and enhanced TG and TC content in the adipocytes. Previous studies have shown that LS intake increases body mass and adiposity in rats and leads to impaired insulin sensitivity [18,29]. The increased body mass in rats fed a LS diet was associated with a lower expression of uncoupling protein 1 (UCP1) in brown adipose tissue and reduced energy expenditure in rats fed the LS chow [30]. It has also been shown that chronic AT1 receptor stimulation impairs adipogenesis, reducing the differentiation of pre-adipose cells into mature adipocytes [31,32] and favoring cellular hypertrophy [33]. We observed increased AT1 expression in periepididymal adipocytes which may help explain the development of insulin resistance that we observed in the present study. Increased adipocyte volume is positively associated with tissue and systemic inflammation (chronic and low-intensity inflammation) and insulin resistance [34,35], which are factors that adversely alter glycolipid metabolism, and we also observed increased adipocyte area in the LS diet groups.

Previous in vitro and in vivo studies show that ANG II stimulates the secreted protein acidic and rich in cysteine (SPARC) [36,37]. SPARC increases collagen synthesis and maturation, and elicits pro-fibrinogenic effects and inhibition of angiogenesis, impairing the differentiation of pre-adipocytes into adipocytes. Besides favoring fibrosis and inflammation, such mechanisms impair the capacity of adipose tissue to store TG, which leads to a predisposition toward ectopic fatty acid deposition in the liver, heart, skeletal muscles, and kidneys and contributes to insulin resistance [37,38,39]. Moreover, AT1 receptor stimulation by ANG II triggers C-terminal-binding protein (CtBP) expression, which inhibits adipocyte differentiation and maturation [39]. Adipocyte size is a critical determinant of adipose tissue function, regardless of the degree of obesity [40,41]. In addition to adipose tissue quantity and distribution, the size of adipose cells is independently associated with insulin resistance [41]. Such mechanisms reinforce the enhanced adiposity observed in sedentary and trained mice that were fed a LS diet. The positive association between plasma angiotensinogen (AGT) concentration and body mass index in different human populations also suggests that increased AGT synthesis by adipose mass may contribute to the increased circulating level of AGT in obesity [42].

Clinical study has also showed a higher concentration of ANG II (23%) in the venous drainage of the abdominal adipose tissue, suggesting that human adipose tissue is a source of ANG II [43]. ANG II increased activity of lipogenic enzymes and selective uptake of esterified cholesterol from HDL [44], which favor TG and esterified cholesterol accumulation in adipocytes and HDL-C reduction [45]. These mechanisms support the increased TG and TC concentrations and enhanced adiposity in the periepididymal adipose tissue, and the reduced HDL-C in mice fed the LS diet observed in our study.

LDLR KO mice, chronically infused for 28 days with ANG II, showed increased AGT mRNA content in periepididymal adipose tissue. The results show that, through an endocrine positive feedback cycle, the increase in systemic ANG II concentration induces AGT expression in adipose tissue, making adipose tissue a prominent site of AGT, which may contribute to RAAS hyperactivity in obesity-associated dysfunctions and plays an important role in the pathogenesis of ANG II-related CVD [46]. Although AT1 receptor mRNA is regulated in a tissue-specific manner [47], the positive feedback loop, in which the increased circulating ANG II concentration promotes AT1 receptor mRNA expression in adipocytes [46] supports the findings of our study, in which mice fed the LS diet expressed higher AT1 receptor content in periepididymal adipose tissue. This mechanism favors the adverse effects resulting from AT1 receptor signaling, such as oxidative insult, inflammation, insulin resistance, adipocyte hypertrophy, visceral adiposity and dyslipidemia, which were evidenced in the present study and contribute greatly to cardiovascular disease.

Thus, clinical and preclinical studies suggest that the increased stimulation of systemic and adipose RAAS system is linked to enhanced adiposity and insulin resistance, and may be a potential causal association for metabolic disorders. It has been proposed that chronic AT1 receptor stimulation decreases lipolysis in adipocytes due to the inhibitory Gi protein activation and, consequently, reduces cAMP synthesis, resulting in limited protein kinase A activation, impaired phosphorylation of hormone-sensitive lipase, and inhibited TG hydrolysis in adipose tissue [35]. Such mechanisms contribute to TG accumulation in adipose tissue, as shown in the present study. In the present study, the LS diet reduced AT1 receptor gene expression (*Agrt1*) and increased the content of AT1 receptor in the periepididymal adipose tissue. This paradoxical finding may be attributed to the complexity of post-transcriptional mechanisms and the substantial differences in the half-lives of proteins in vivo, making it difficult to calculate protein concentrations from mRNA [48]. Thus, the association between protein concentrations and their respective mRNAs is often not considered strong, and gene transcription and translation generally do not present a linear and simple relationship [49,50].

Hypertrophic adipocytes secrete pro-inflammatory molecules such as monocyte chemoattractant protein-1, interleukin (IL)-6, IL-8, and leukotrienes, demonstrating a pro-inflammatory profile compared to eutrophic adipocytes [51]. These factors, associated with increased NEFA concentration, promote recruitment of pro-inflammatory macrophages, subtype M1, and reduce the population of anti-inflammatory macrophages, subtype M2 [52]. In current study, we showed that the LS diet increased the content of CD11b (marker of pro-inflammatory macrophages, subtype M1) and reduced the expression of CD206 (marker of anti-inflammatory macrophages, subtype M2) in periepididymal adipocytes.

Pro-inflammatory cytokines, secreted by macrophages from hypertrophied adipose tissue, have paracrine and endocrine effects that reduce insulin sensitivity. Activation of tissue macrophages promotes the release of chemokines, which recruit additional macrophages, increasing the macrophage content in adipose tissue, and diffuse chronic inflammation. In insulin target cells, tumor necrosis factor-alpha, IL-6, IL-1beta, together with other cytokines and factors secreted by macrophages, activate JNK and nuclear factor kappa-B inhibitory kinase beta subunit. These protein kinases activate the nuclear factor kappaB, promote the transcription of pro-inflammatory genes, and the phosphorylation of serine residues in insulin receptor, IRS and other insulin signaling molecules, implying insulin resistance [53]. Thus, our results suggest that the adverse macrophage polarization in periepididymal adipose tissue promoted by the dietary sodium restriction may contribute to insulin resistance and hyperlipidemia. 

In summary, although the relevance of our findings must be confirmed in humans, our results robustly demonstrate that AET protected against insulin resistance but not against inflammation and the increase in visceral adiposity in mice fed the LS diet. These results highlight how chronic and intense dietary sodium restriction adversely modulates the glycolipid metabolism in visceral adipose tissue and the importance of visceral adipose tissue as a target for pharmacological and non-pharmacological therapeutic interventions oriented toward improving cardiometabolic diseases.

The present study also has its limitations. Although we demonstrate new systemic and visceral adipose tissue changes in glycolipid metabolism, the intracellular signaling pathways that promote metabolic impairment were not investigated. In addition, the identification and quantification of lipids by lipidomics analysis in adipose tissue and the evaluation of adipocyte number, volume, and surface area size using a particle size analyzer [54] would make it possible to more accurately associate the LS diet induced-changes in the adipose tissue with CVD. The acute glucose uptake in the ITT depends a lot on skeletal muscle tissue. Since we did not assess total lean mass, skeletal muscle capillarization, blood flow, and modulation of skeletal muscle energy substrate metabolism in the mice, we do not know whether skeletal muscle metabolism differed among the groups of mice, which could have some influence on the ITT. 

Taken together, our results showed that, in LDLR KO mice, dietary sodium restriction increased body mass, glycemia, and insulin resistance, and adversely altered lipid metabolism by increasing triglyceridemia, LDL-C, and VLDL-TG and reducing HDL-C and HDL-TG, showing a pro-atherogenic lipid profile. The LS diet markedly increased visceral adiposity, individual adipocyte area, and the content of TC and TG in the periepididymal adipose tissue. Furthermore, the LS diet stimulated infiltration of pro-inflammatory macrophages (M1 subtype) and reduced the population of anti-inflammatory macrophages (subtype M2) in the periepididymal adipose tissue. Among the evaluated parameters, AET only protected against peripheral insulin resistance.

## 4. Materials and Methods

### 4.1. Animal 

LDLR KO mice on a C57BL/6J background were sourced from the Jackson Laboratories (Bar Harbor, ME, USA) and bred in our animal facility at a room temperature of 22 ± 2 °C and a 12 h light/dark cycle. The mice had free access to pelleted commercial chow (Nuvilab CR1— Quimtia S/A, Colombo, PR, Brazil) and drinking water until the beginning of the experimental protocols. The experimental protocols involving the animals were approved by the Animal Care and Research Advisory Committee of the Faculdade de Medicina da Universidade de Sao Paulo (CEUA # 148/16) and carried out according to the U.S. National Institutes of Health Guide for the Care and Use of Laboratory Animals.

### 4.2. Experimental Protocols

At 12 weeks of age, male LDLR KO mice were fed ad libitum either a normal-sodium diet (NS; 0.5% sodium = 1.27% NaCl; Envigo Teklad Diets—Indianapolis, IN, USA—TD92140) or a low-sodium diet (LS; 0.06% sodium = 0.15% NaCl; Envigo Teklad Diets— Indianapolis, IN, USA—TD 92141). Each diet contained approximately the following nutrients (g/100 g): casein (28.7); sucrose (31.3); cornstarch (20.0); soybean oil (6.0); minerals; and vitamins. The concentration of sodium chloride in the LS diet was sufficient to provide enough sodium and chloride for normal body development in rodents [55].

Before distributing animals into the different diet groups, mice were matched to avoid baseline differences in key parameters, such as maximum exercise capacity, body mass (BM), hematocrit, systolic BP (SBP), fasting blood glucose, total cholesterol (TC), and TG levels, to assure homogeneity among the experimental groups. The mice were then assigned to four experimental groups (*n* = 20 mice/group) according to diet sodium concentration and aerobic exercise training (AET) or sedentarism: (1) mice fed the NS diet and kept sedentary (NS–S); (2) the mice fed the NS diet and trained (NS–T); (3) the mice fed the LS diet and kept sedentary (LS–S); or (4) mice fed the LS diet and trained (LS–T). Each animal group inside the cage was considered an experimental unit. Thus, the BM of each animal and diet intake per group were weekly assessed during the 90-day experimental protocol. Plasma TC, TG, and glucose levels, as well as hematocrit, maximum exercise capacity, and SBP were measured again at the end of the 13 weeks experimental protocol. An insulin tolerance test (ITT), 24-h urinary sodium excretion (U_Na_), and LP profile were also determined at the end of the experimental protocol. The experimental protocol was in accordance with the ethical precepts for reducing the number of animals in scientific research, since part of the results presented in the current study was previously published by our group using the same mice [15,16,17]. Mice were anesthetized with sodium thiopental (THIOPENTAX^®^) by i.p. injection (150 mg/kg of BM) and periepididymal adipose tissue was excised and used for lipid, protein, gene transcription, and histopathological analysis. 

### 4.3. Blood Sampling, Glucose, and Biochemical Analyses of Plasma

After a 12 h fasting period, blood samples (200 µL) from tail veins were collected into heparinized microhematocrit capillary tubes. Plasma TC and TG concentrations were assessed using colorimetric enzymatic kits (Labtest do Brasil, Lagoa Santa, MG, Brazil), and blood glucose concentration was measured using Accu Check^®^ Performa glucometer and strips (Roche, Sao Paulo, SP, Brazil). Hematocrit was determined by the microhematocrit method after centrifuging at 12,000 rpm for × 30 min.

### 4.4. Lipoprotein Profile

Serum lipid profiling (e.g., very low-density lipoprotein—VLDL, LDL, and HDL) was analyzed by fast protein liquid chromatography (FPLC) at the end of experimental protocol. Plasma samples (100 µL) were injected into Superose HR 10/30 6 column (FPLC System, Pharmacia, Upsalla, Sweden) and eluded in constant flow of 0.5 mL/min, with Tris buffer (Tris 10 nM, NaCl 150 mM, EDTA 1 mM, and NaN_3_ 0.03%—pH 7.2). The LP cholesterol and TG contents were determined by enzymatic colorimetric kits (Labtest do Brasil, Lagoa Santa, MG, Brazil).

### 4.5. Blood Pressure Measurement

SBP was measured by the non-invasive tail-cuff plethysmography using Visitech Systems (model BP-2000-M2 Blood Pressure Analysis System- Visitech Systems, Inc. Apex, NC, USA) after the mice were preconditioned to the instrument. Eight readings were recorded in two consecutive days and averaged to obtain the mean values.

### 4.6. Insulin Tolerance Test

The mice were fasted for 4 h and received a single intraperitoneal injection of insulin (1 U/kg of BM; Humulin R, Eli Lilly, Sao Paulo, SP, Brazil). Blood samples were drawn from the tail vein for determination of blood glucose using Accu Check^®^ Performa glucometer and strips (Roche, Sao Paulo, SP, Brazil) at times 0 (before injection), 10, 20, and 30 min after insulin injection. The blood glucose decay rate (kITT) was determined by linear regression between baseline and 30 min mark [56,57].

### 4.7. Aerobic Exercise Training

Animals were submitted to the AET protocol 5 days per week, at 15 m/min, 60 min per day, for 90 days using a treadmill specific for mice (WEG, São Carlos, SP, Brazil) [58]. Animals that did not acclimatize to running on the treadmill were excluded from the study (~10% of the mice). The maximum exercise capacity test was performed as previously described [59]. Briefly, an initial speed of 9 m/min, 0% inclination, and increases of 3 m/min every 3 min until complete inability to run were the parameters used for the maximum exercise capacity test. The maximum running time of each animal was used as the reference of physical conditioning.

### 4.8. Adipose Tissue Analysis 

After euthanasia, the periepididymal adipose tissue was removed and subsequently washed with ice-cold 0.9% sodium chloride solution and divided into two sections; one section was frozen for mRNA analysis and measurement of TG, TC, and protein, while the other section was immersed in a buffered formaldehyde fixative solution (10%) for 24 h. The fixed portion of periepididymal fat was then transferred to a 70% ethanol solution for 48 h and embedded in paraffin for histomorphometric, immunofluorescence, and immunohistochemistry analysis. Four-μm-thick cross-sections were assigned to hematoxylin–eosin (H&E) staining [60] and morphometric analysis. Digital images were scanned using 200× magnification and analyzed with the Image-Pro Plus 6.0^®^ program. To assess the percentage of intercepts and the area of adipocytes, the Gundersen stereological method [61] was used, consisting of point counting using a reticulum formed by 100 points (area: 62,500 µm^2^) distributed orthogonally over the image. The percentage of intercepts was calculated from the intercepts that hit the pericellular connective tissue by the total number of points that hit the surface of the adipose tissue, multiplied by 100; the adipocyte area was then inferred using the following calculation: 0.625 µm^2^/no of intercepts.

### 4.9. Western Blotting

Protein kinase B (AKT), phosphorylated AKT (pAKT), and glucose transporter type-4 (GLUT4) content in the periepididymal adipose tissue were determined by western blotting. Briefly, 30 μg of total adipose tissue homogenate was submitted to electrophoresis into a 10% polyacrylamide gel and after transference to a nitrocellulose membrane, immunoblot was performed by using total and pAKT antibody (1:1000—Sc-8312 and Sc-7985-R, Santa Cruz Biotechnology, Dallas, TX, USA) and GLUT4 antibody (1:3000—07-1404, Milipore Corporation, Burlington, MA, USA) primary antibodies. After incubation with secondary anti-rabbit antibody conjugated to peroxidase (1:5000), membranes were incubated with enhanced chemiluminescence reaction. The Ponceau staining of GLUT4, pAKT, and AKT were utilized to normalize the optical density of each band. Results were presented in arbitrary units corrected per gram of tissue.

### 4.10. Gene Expression

Total RNA was extracted from the periepididymal adipose tissue by Trizol reagent (Invitrogen Life Technologies, Carlsbad, CA, USA) and purified with the RNeasy Mini Kit (Qiagen, Germantown, MD, USA). Total RNA (2 μg) was reverse transcribed to cDNA using a High Capacity RNA-to-cDNA kit (Applied Biosystems, Foster City, CA, USA) according to the manufacturer’s instructions. Real-time quantitative PCR (Applied Biosystems, CA, USA) was performed at the following temperatures: holding stage (50 °C by 2 min and 95 °C by 10 min) and 40 amplification cycles (95 °C by 15 s and 60 °C by 1 min) by Taqman assays (Applied Biosystems). The following TaqMan Gene Expression Assays were used: *Ccl2* (Chemokine (C-C motif) ligand 2, Mm00441242_m1), *Adipoq* (Adiponectin, Mm01343606_m1), *Itgam* (Integrin alpha M, Mm00434455_m1), *Mrc1* (Mannose receptor, C type-1, Mm01329362_m1), *Retn* (Resistin, Mm00445641_m1), *Slc2a4* (Solute carrier family 2 facilitated glucose transporter, member 4, Mm00436615_m1), and *Agtr1a* (Angiotensin II receptor, type-1a, Mm01166161_m1) in the StepOne Plus—Real-Time PCR System (Applied Biosystems by Life Technologies). The relative expression of each gene was normalized to the housekeeping gene; *Ppib* (Peptidylprolyl isomerase B, Mm00478295) and relative quantification analysis was performed with the StepOne Software 2.0 (Ap Applied Biosystems, Foster City, CA, USA) using the comparative cycle threshold (Ct) (2^−ΔΔCt^) method [62].

### 4.11. Biochemical Analysis of Adipose Tissue

Lipid extraction was performed according to Folch [21]. Briefly, 100 mg of adipose tissue was macerated in a solution of chloroform:methanol (2:1, *v*:*v*) and samples were maintained overnight at −20 °C. The organic phase was separated adding aqueous solution containing 0.05% H_2_SO_4_ following centrifugation (1690× *g*). The solvent was dried Genivac Standard EZ-2 (Genivac, Huntington Station, NY, USA), added with 1 mL Triton X-100 solution (0.5% in chloroform) and dried again Genivac Standard EZ-2 (Genivac, Huntington Station, NY, USA). The samples were resuspended in 500 µL of water and heated at 37 °C for 15 min with agitation. TG and TC were determined by enzymatic colorimetric kits (Labtest, Lagoa Santa, MG, Brazil) in automated equipment COBAS-MIRA (Roche Diagnostics, Indianapolis, IN, USA). TC and TG concentration were corrected by total mass of periepididymal adipose tissue.

### 4.12. Immunohistochemistry

Subpopulations of macrophages were identified by immunohistochemistry. The histological sections were deparaffinized and a 0.3% hydrogen peroxide solution was applied during for 4–5 min to inhibit endogenous peroxidase activity. Tissue sections were pretreated in citrate buffer solution, pH 6.0, and heated in a Pascal pressure cooker (125 °C, for 1 min) to unmask the epitopes. The primary antibodies for immunohistochemical reactions were purchased from Abcam (Cambridge, Cambridgeshire, UK) and consisted of anti-CD11b (Cat. # ab64347; 1:200) and anti-CD206 (Cat. # ab64693; 1:1250). The reaction was revealed using biotin–streptavidin–peroxidase (Peroxidase, Mouse IgG; PK6102) VECTASTAIN Elite ABC HRP Kit (Vector Laboratories, Burlingame, CA, USA), according to the manufacturer’s instructions. The 3,3 diaminobenzidine (Sigma Chemical, St Louis, MO) was used as a chromogen. The sections were counterstained with Harris hematoxylin (Merck, Darmstadt, HE Germany). For negative controls, the primary antibody was replaced by phosphate-buffered saline (PBS). Immunostaining was assessed by the stereological point-counting method, consisting of a reticle made up of 100 points [61] and analyzed using the Image-Pro Plus 6.0 system composed on an Olympus camera (Olympus Co, St Laurent, QC, Canada) coupled to an Olympus microscope (Olympus BX51). From here, the images were sent to an LG monitor by means of a digitizing system (Oculus TCX, Coreco, Inc, St. Laurent, QC, Canada) and downloaded to a computer Pentium 1330 Mhz (Intel, Santa Clara, CA, USA). The percentage (P) of points marked in the reference compartment for each antibody was expressed according to the formula: P = (Pi × 100)/Pt, where Pi corresponded to the number of points that were positively marked by immunohistochemistry and Pt to the total number of points analyzed. The percentage (P) of each antibody was considered to be the average of the sum of the results from all the histological fields analyzed for each sample.

### 4.13. Immunofluorescence Staining

Histological sections were incubated for 24 h at 4 °C with rabbit polyclonal antibody anti-angiotensin II type-1 (AT1) receptor (1:30; Santa Cruz Biotechnology, Dallas, TX, USA) diluted in PBS. Samples were washed in PBS and incubated for 60 min at room temperature with Alexa Fluor^®^ 488-conjugated goat anti-rabbit IgG antibody (Invitrogen, Fisher Scientific Baltics UAB, Cat # A11008, Vilnius, Lithuania) diluted at 1:200 in a PBS solution containing 0.003% Evans blue dye. After washing with PBS, the sections were incubated for 5 min at room temperature with 4,6-diamidino-2-phenylindole, dihydrochloride (DAPI) diluted at 1:200 in PBS.

### 4.14. Statistical Analyses

Statistical analysis was performed using the Minitab 19 software (Minitab LL, Stage College, PA, USA). Data normality was checked by the Kolmogorov–Smirnov test, non-normal variables were Johnson-transformed before analysis of variance. Differences at the baseline were compared by one-way ANOVA. Variables with non-normal distribution were transformed using Johnson’s method before an analysis of variance or Kruskal–Wallis test. Results after intervention were compared by two-way ANOVA (generalized linear model) with fixed factors: diet and exercise. Tukey’s post-test was applied when appropriate. In order to verify pre- and post-effects, the analyses of exercise capacity and food consumption were assessed using repeated measures ANOVA (mixed model; random factor: animal, and fixed factors: diet, exercise, and time). The statistical differences could be indicated by the effect of the diet (NS vs. LS), exercise (S vs. T), or the interaction. When the interaction was reached, distinct letters were used to represent statistical differences among groups (*p* < 0.05). Data are presented as mean ± standard deviation (SD) and median (periepididymal adipose tissue mass).

## 5. Conclusions

Our results showed that, in LDLR KO mice, dietary sodium restriction increases body mass, glycemia, and insulin resistance, and adversely alteres lipid metabolism by increasing triglyceridemia, LDL-C, and VLDL-TG, and reducing HDL-C and HDL-TG, showing a pro-atherogenic lipid profile. The LS diet markedly increased visceral adiposity, individual adipocyte area, and the content of TC and TG in the periepididymal adipose tissue. Furthermore, the LS diet stimulated an infiltration of pro-inflammatory macrophages (M1 subtype) and a reduced population of anti-inflammatory macrophages (subtype M2) in the periepididymal adipose tissue. Among the evaluated parameters, AET only protected against peripheral insulin resistance.

## Figures and Tables

**Table 1:** Baseline body mass, biochemical profile, hematocrit, and systolic blood pressure of LDL receptor knockout mice before starting the normal-sodium (NS) or the low-sodium (LS) diet or aerobic exercise protocols.

	NS-S	NS-T	LS-S	LS-T	*p*
BM (g) (*n* = 15, 12, 14, 12)	24 ± 2	25 ± 1	25 ± 2	24 ± 2	0.76
Glucose (mmol/L) (*n* = 15, 12, 15, 12)	5.2 ± 0.7	5.2 ± 0.4	5.2 ± 0.8	5.3 ± 0.8	0.93
Hematocrit (%) (*n* = 15, 12, 15, 11)	49 ± 5	49 ± 7	49 ± 7	51 ± 5	0.15
TC (mmol/L) (*n* = 4, 3, 5, 3)	6.9 ± 0.9	6.6 ± 0.9	7.4 ± 0.9	6.8 ± 1.0	0.37
TG (mmol/L) (*n* = 4, 3, 5, 3)	1.9 ± 0,4	1.7 ± 0.3	1.8 ± 0.4	1.7 ± 0.3	0.87
SBP (mmHg) (*n* = 13, 10, 14, 12)	109 ± 8	113 ± 6	111 ± 6	111 ± 5	0.44

BM, body mass; systolic blood pressure, SBP; TC, total cholesterol; TG, triglycerides. Results are expressed as mean ± standard deviation (SD) and compared by one-way ANOVA with Tukey’s post-test. *n* = number of mice.

**Table 2:** Body mass, biochemical profile, hematocrit, and systolic blood pressure of LDL receptor knockout mice fed either a normal-sodium (NS) or a low-sodium (LS) diet, sedentary (S) or trained (T), at the end of the 90-day experimental protocol.

	NS-S	NS-T	LS-S	LS-T	Diet (*p*)	AET (*p*)	Interaction (*p*)
BM (g) (*n* = 15, 12, 14, 12)	26 ± 2.6	25 ± 1.5	28 ± 3.0	27 ± 2.4	0.008	-	-
Glucose (mmol/L) (*n* = 15, 12, 15, 12)	5.6 ± 0.8	6.2 ± 1.1	6.4 ± 1.0	6.9 ± 1.3	0.01	-	-
Hematocrit (%) (*n* = 15, 12, 15, 11)	48 ± 3.2	49 ± 2.7	49 ± 3.6	50 ± 4.7	-	-	-
TC (mmol/L) (*n* = 4, 3, 5, 3)	7.0 ± 2.6	8.7 ± 2.6	7.7 ± 3.3	7.5 ± 2.1	-	-	-
TG (mmol/L) (*n* = 4, 3, 5, 3)	1.1 ± 0.1	1.2 ± 0.4	1.8 ± 0.5	1.5 ± 0.6	0.049	-	-
SBP (mmHg) (*n* = 13, 10, 14, 12)	106 ± 6.1	105 ± 4.9	104 ± 7.6	103 ± 4.8	-	-	-
U_Na_ (mEq/24 h) (*n* = 14, 12, 13, 12)	0.2 ± 0.10	0.2 ± 0.1	0.04 ± 0.02	0.03 ± 0.02	<0.001	-	-

BM, body mass; SBP, systolic blood pressure; TC, total cholesterol; TG, triglycerides; U_Na_, 24-h urinary sodium excretion. Results are expressed as mean ± standard deviation (SD) and compared by two-way ANOVA with Tukey’s post-test. *n* = number of mice.

## Data Availability

All data reported are included in the manuscript and upon personal request to the authors raw data can be shared. This article includes original data and only kITT and biochemical data from the LS and the NS groups were shared with another previous publication as informed.
